# Synthesis, Docking, and DFT Studies on Novel Schiff Base Sulfonamide Analogues as Selective COX-1 Inhibitors with Anti-Platelet Aggregation Activity

**DOI:** 10.3390/ph17060710

**Published:** 2024-05-30

**Authors:** Yasmine M. Abdel Aziz, Mohamed S. Nafie, Pierre A. Hanna, Sherif Ramadan, Assem Barakat, Marwa Elewa

**Affiliations:** 1 Pharmaceutical Organic Chemistry Department, Faculty of Pharmacy, Suez Canal University, Ismailia 41522, Egypt; marwa_elewa@pharm.suez.edu.eg; 2 Department of Chemistry, College of Sciences, University of Sharjah, Sharjah P.O. Box 27272, United Arab Emirates; mohamed.elsayed@sharjah.ac.ae; 3 Chemistry Department, Faculty of Science, Suez Canal University, Ismailia P.O. Box 41522, Egypt; 4 Department of Pharmaceutics and Industrial Pharmacy, Faculty of Pharmacy, Suez Canal University, Ismailia 41522, Egypt; pierre_hanna@pharm.suez.edu.eg; 5 Chemistry Department, Michigan State University, East Lansing, MI 48824, USA; sramadan@chemistry.msu.edu; 6 Department of Chemistry, College of Science, King Saud University, P.O. Box 2455, Riyadh 11451, Saudi Arabia

**Keywords:** sulfonamide, Schiff base, COX-1, COX-2, anti-platelet aggregation, DFT

## Abstract

Selective COX-1 inhibitors are preferential therapeutic targets for platelet aggregation and clotting responses. In this study, we examined the selective COX-1-inhibitory activities of four newly synthesized compounds, **10**–**13**, along with their abilities to inhibit platelet aggregation against ADP and collagen. The target compounds **10**–**13** were synthesized using the conventional method, sonication, and microwave-assisted methods. Microanalytical and spectral data were utilized to elucidate the structures of the new compounds **10**–**13**. Additionally, a spectral NMR experiment [NOESY] was conducted to emphasize the configuration around the double bond of the imine group C=N. The obtained results revealed no observed correlation between any of the neighboring protons, suggesting that the configuration at the C=N double bond is *E*. Biological results revealed that all the screened compounds **10**–**13** might serve as selective COX-1 inhibitors. They showed IC_50_ values ranging from 0.71 μM to 4.82 μM against COX-1 and IC_50_ values ranging from 9.26 μM to 15.24 μM against COX-2. Their COX-1 selectivity indices ranged between 2.87 and 18.69. These compounds show promise as promising anti-platelet aggregation agents. They effectively prevented platelet aggregation induced by ADP with IC_50_ values ranging from 0.11 μM to 0.37 μM, surpassing the standard aspirin with an IC_50_ value of 0.49 μM. Additionally, they inhibited the platelet aggregation induced by collagen with IC_50_ values ranging from 0.12 μM to 1.03 μM, demonstrating superior efficacy compared to aspirin, which has an IC_50_ value of 0.51 μM. In silico molecular modeling was performed for all the target compounds within the active sites of COX-1 and COX-2 to rationalize their selective inhibitory activities towards COX-1. It was found that the binding interactions of the designed compounds within the COX-1 active site had remained unaffected by the presence of celecoxib. Molecular modeling and DFT calculations using the B3LYP/6-31+G (d,p) level were performed to study the stability of *E*-forms with respect to *Z*-forms for the investigated compounds. A strong correlation was observed between the experimental observations and the quantum chemical descriptors.

## 1. Introduction

Cyclooxygenase (COX) serves as the pivotal enzyme in the rate-limiting step in prostanoid biosynthesis. Prostanoids, which act as autocoid mediators, play crucial roles in physiological and pathological processes. Cyclooxygenase (COX) comprises identical homodimers subunits: cyclooxygenase-1 (COX-1) and cyclooxygenase-2 (COX-2) [1,2,3]. During catalysis, it has been found that only one subunit is activated at a time. COX-2 is notably implicated in the pathophysiology of inflammation and tumorigenesis [4,5] while COX-1 primarily regulates platelet functions. It facilitates platelet activation, adhesion, and aggregation, thereby contributing the clotting response [6,7,8]. 

**Figure 1:**
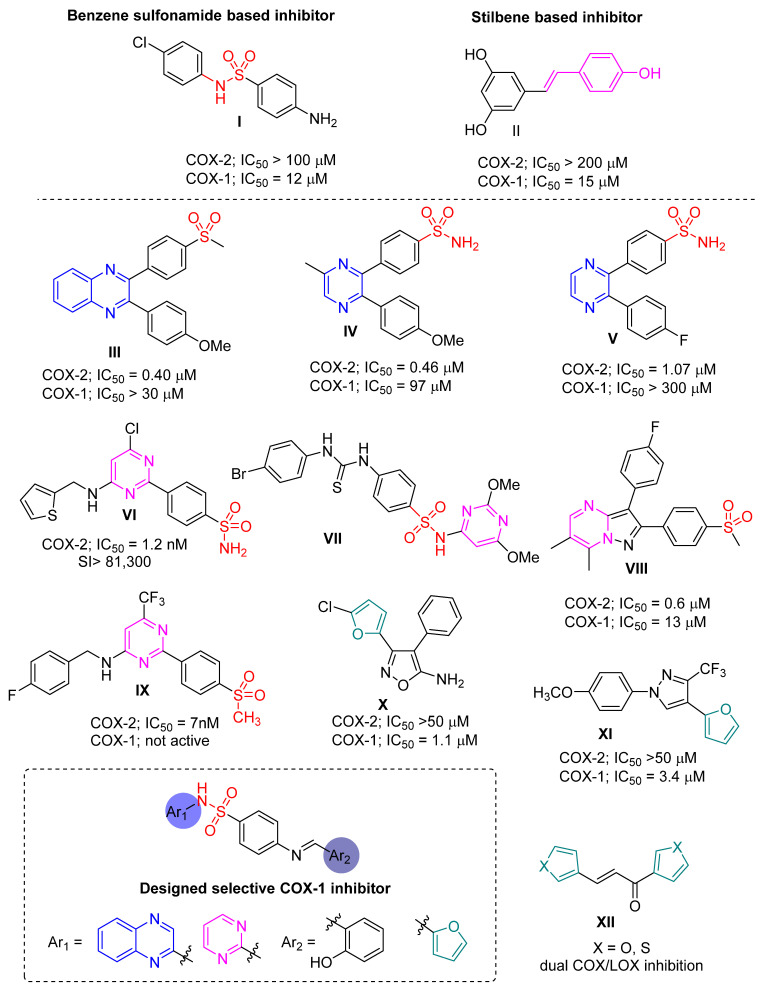
The design of selective COX-1 inhibitors based on previous selective examples having different pharmacophoric groups.

Coxibs are widely used anti-inflammatory drugs and are renowned for their selective inhibition of COX-2. Surprisingly, it has been found that coxibs can tightly bind to COX-1 active sites and impede the binding interactions of COX-1 inhibitors like aspirin. Aspirin, known to inhibit both COX-1 and COX-2, carries a toxic acetyl tail that acetylates the serine residue of COX-1 [9]. This acetylation leads to permanent damage to the COX-1 active site. COX-2 is inhibited by the same mechanism; however, higher doses of aspirin are required for effective COX-2 inhibition. Moreover, several studies have suggested that lower doses of aspirin are adequate for inhibiting platelet COX-1. However, a significant limitation of aspirin use is that coxibs interfere with its anti-platelet aggregation effect when administered concurrently [10,11,12]. These findings have prompted us to develop novel COX-1 inhibitors, distinct from aspirin, capable of controlling platelet aggregation even in the presence of coxibs. Another limitation is the scarcity of studies focusing on the design of selective COX-1 inhibitors. From the available research, we have selected two promising selective COX-1 inhibitors to serve as models in the design of our target compounds. Compound **I** is a benzene sulfonamide derivative with an IC_50_ value of 12 μM against COX-1 and a selectivity index of approximately 100 folds. Compound **II** is a stilbene derivative with an IC_50_ value of 15 μM against COX-1 and a selectivity index of around 200 folds [13]. Drawing from the mixed pharmacophore theory [14], novel Schiff base analogues were designed based on two pharmacophoric regions (Figure 1). Region I is a benzene sulfonamide moiety inspired by the structure of compound I. Region II is a stilbene-like Schiff base moiety akin to compound **II**. 

Heterocycles like pyrimidine, quinoxaline, and furan [15,16,17,18,19,20] are crucial in developing selective cyclooxygenase (COX) inhibitors, which are essential in medicinal chemistry for managing inflammation and pain. COX-2 is a primary target, and selective inhibitors aim to minimize side effects. Pyrimidine derivatives selectively inhibit COX-2 while sparing COX-1, reducing gastrointestinal risks. Quinoxalines exhibit potent anti-inflammatory properties and are investigated for COX inhibition. Furan derivatives also show promise in preclinical COX inhibition studies. These heterocyclic structures improve selectivity and aid in optimizing drug properties, advancing the development of safer and more effective anti-inflammatory drugs. As representative examples, compounds **III**–**V** as quinoxaline derivatives have shown selective activity against COX-2 rather than COX-1. Also, pyrimidine scaffold has exhibited good potential against COX enzymes such as compounds **VI**–**IX**, with these compounds being mainly more active towards COX-2. Also, it has been found that the insertion of the furan moiety leads to a good enhancement in reactivity towards the COX-1 enzyme; for example, compounds **X** (COX-1; IC_50_ = 1.1 μM) [21], **XI** (COX-1; IC_50_ = 3.4 μM) [22], and **XII** have been reported as dual inhibitors for COX/LOX enzymes [23]. To explore the combination of the different pharmacophoric groups into one compound is a challenge. In this text we have designed, synthesized, and evaluated a novel selective COX-1 inhibitor having the pyrimidine, quinoxaline, and furan motifs for the first time.

It is worth noting that recently, in the field of drug discovery, computational chemistry has progressed in correlating the experimental observations and the theoretical data of new candidates [24]. The density functional theory (DFT) is one of the most important theoretical methods that are used in calculating a great variety of molecular properties [25,26,27,28]. The aim of this study was to utilize DFT to investigate the difference in energy between the *E*- and *Z*-forms of novel Schiff base analogues. These analogues might selectively recognize the platelet COX-1 active site.

## 2. Results and Discussion

### 2.1. Chemistry

**Scheme 1:**
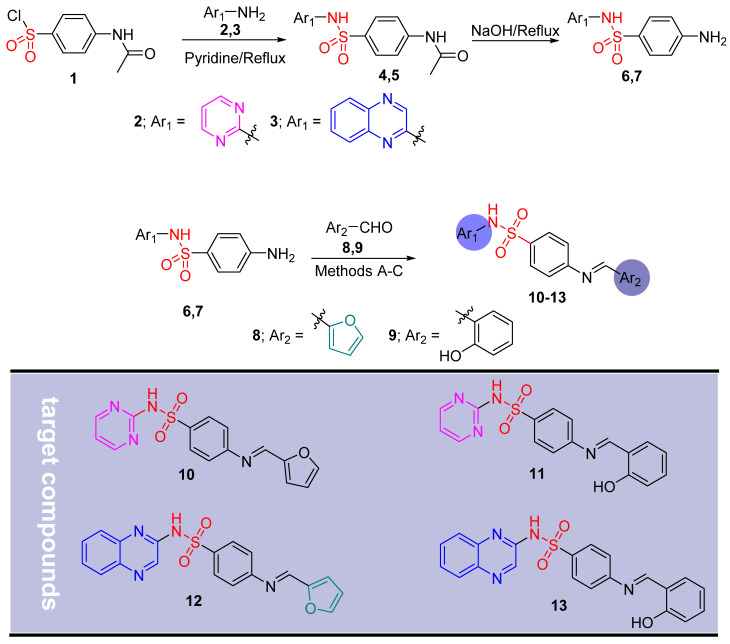
Synthesis of target compounds **10**–**13**.

Scheme 1 illustrates the synthetic route developed for the preparation of four new Schiff base sulfonamide analogues, designated as **10**–**13**. The biological significance of Schiff base analogues has motivated numerous researchers to devise various methods for their synthesis [29]. In this study, our objective was to synthesize the target compounds **10**–**13** through the conventional method, sonication, and microwave-assisted methods [30,31,32,33].

Microanalyses and analyses of spectral data (IR, ^1^H NMR,^13^CNMR, and EI-MS) were carried out to confirm the chemical structures of the new compounds **10**–**13**. The IR spectra displayed NH stretching at around 3400–3500 cm^−1^ and bending at around 1550 cm^−1^, the appearance of O=S=O asymmetric stretching at around 1350 cm^−1^ and symmetric stretching at around 1140 cm^−1^, and the appearance of =C-H stretching at around 2900 cm^−1^. There was also an appearance of C=N at around 1650 cm^−1^. Additionally, the IR spectra of compounds **11** and **13** showed O-H stretching at around 3150 cm^−1^. ^1^H-NMR spectra of the target compounds **10**, **11**, **12**, and **13** showed NH singlets at δ_H_ [11.30–11.68 ppm] in addition to aromatic multiplets at δ_H_ [6.80–8.20 ppm]. Regarding the ^1^H NMR spectra of compounds **11** and **13**, singlets at δ_H_ [12.50 ppm] were detected that referred to the hydroxyl group (OH). 

An additional spectral NMR experiment, specifically a NOESY (Nuclear Overhauser Effect Spectroscopy), was conducted to highlight the configuration around the double bond of the imine group (C=N). The obtained results revealed no observed correlation between any of the neighboring protons in the space, suggesting that the configuration at the C=N double bond is *E*. (Supplementary Materials).

### 2.2. Biological Activity

#### 2.2.1. Cyclooxygenase Inhibition Assays

The cyclooxygenase-inhibitory activities of the four newly compounds **10**–**13** were investigated against COX-1 and COX-2. The COX-1 selectivity indices were also calculated. The results are listed in Table 1.

The tested compounds displayed IC_50_ values ranging from 0.71 to 4.82 μM against COX-1 and IC_50_ values ranging from 9.26 to 15.24 μM against COX-2. Their selectivity indices for COX-1 ranged between 2.87 and 18.69. Fortunately, it was found that the designed compounds showed, collectively, a selective COX-1 inhibition range superior to the celecoxib for standard compounds I and II. Compound **12** was identified as the most active sulfonamide analogue among the designed compounds with 20, 16.9, and 21 folds compared to the reference (the celecoxib, standard compounds I and II). The chemical structure of compound **12,** having a quinoxaline/furan scaffold-based Schiff base sulfonamide, was the best in the reactivity order **12** > **10** > **13** > **11**. Therefore, the compounds could be considered promising lead molecules and selective COX-1 inhibitors.

#### 2.2.2. The Anti-Platelet Aggregation Assay

Furthermore, the target compounds **10**–**13** were capable of inhibiting the platelet aggregation induced by 10 μM ADP and 0.33 U/mL collagen. The platelet aggregation inhibition rates and the corresponding IC_50_ values are listed in Table 2.

Compounds **10**–**13** showed promising anti-platelet aggregation activity. The IC_50_ ranges against ADP were from 0.11 to 0.37 μM, and against collagen, they were from 0.12 to 1.03 μM. These ranges were superior to the standard aspirin with IC_50_ = 0.49 μM against ADP and IC_50_ = 0.51 μM against collagen. The compound **12,** having quinoxaline and furan moieties, showed the best reactivity with 4.45 folds compared to aspirin in the case of ADP; also, on the other hand, it exhibited more activity than aspirin with 4.25 folds in the case of collagen. The second compound that showed better reactivity was compound **11,** in the case of ADP; the compound had, in its chemical structure, pyrimidine and 2-hydroxy-susbitituted benzene. The third compound in the reactivity order against ADP was **10,** having pyrimidine and a furan nucleus. In the case of collagen, the reactivity order was as follows: **12** > **10** > **11**. Compound **13**-based sulfonamide, having the quinoxaline and 2-hydroxy-susbitituted benzene, was not active in both ADP and collagen.

### 2.3. In Silico Studies

#### 2.3.1. Molecular Docking 

**Figure 2:**
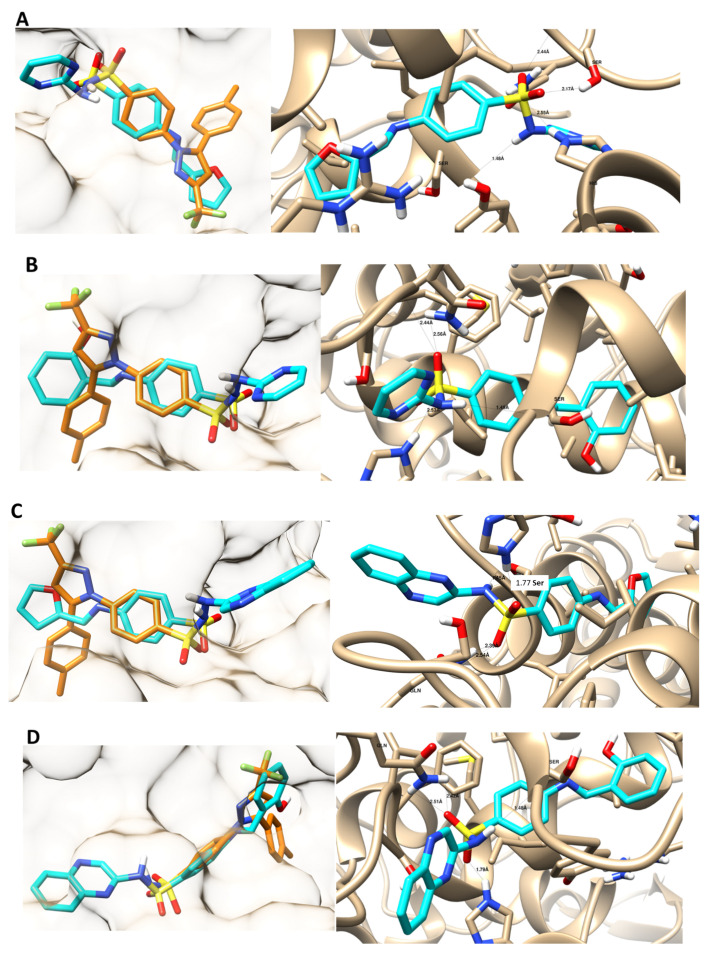
Binding dispositions of the co-crystallized ligand: celecoxib (orange-colored) and the docked compounds (cyan-colored) towards platelet COX-1 active site. (**A**): **10**, (**B**): **11**, (**C**): **12**, and (**D**): **13**. The panel on the left shows the surface representation while the right panel shows the interactive binding mode with the main amino acids.

To rationalize the selectivity of the investigated compounds towards COX-1, the novel compounds were screened for their binding abilities towards the cylooxygenase binding sites (COX-1 and COX-2), highlighting both binding interactions and binding energies (Table 3). The tested compounds were docked inside the COX-1 active site with good binding energies ranging from −16.44 Kcal/mol to −20.70 Kcal/mol (Figure 2). Interestingly, the binding interactions of the target compounds were not hindered by celecoxib. They formed four strong hydrogen bond interactions with the key amino acids Gln 192, His 90, Ser 353, and Ser 516 within the platelet COX-1 active site. The binding energy of the most active compound **12** was −20.70 Kcal/mol, which was superior to the binding energy of celecoxib, −16.91 Kcal/mol. 

Celecoxib as a selective COX-2 inhibitor formed three hydrogen bonds with the key amino acids Leu 338, Gln 178, and Ser 339 within COX-2 active sites with a binding energy of −14.45 Kcal/mol. Unlike celecoxib, compound **10** formed one hydrogen bond with Ser 516 with a binding energy of −9.87 Kcal/mol. Compound **11** formed binding interactions with Arg 499 and His 75 with a binding energy of −8.94 Kcal/mol. Compound **12** formed one hydrogen bond and an Arene-cation with Arg 499 and one hydrogen bond with His 75 with a binding energy of −5.89 Kcal/mol. Compound **13** formed two hydrogen bonds with Arg 106 and Gln 178 with a binding energy of −8.84 Kcal/mol.

To sum up, the target compounds extended deeply in the characteristic pocket of the platelet COX-1 receptor cavity formed by Gln 192, His 90, Ser 353, and Ser 516. Their binding interactions were not hindered by celecoxib. 

Docking calculation was validated using the AutoDock Vina 1 software. The RMSD was lower than 2.

#### 2.3.2. Bioinformatics Study

Bioinformatics studies were also performed to investigate the physicochemical properties and drug-like properties of compounds **10**–**13**. 

**Figure 3:**
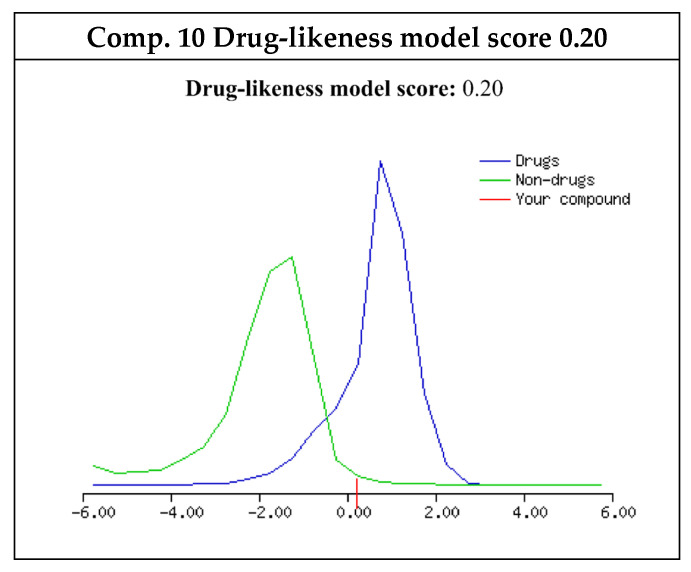
Plots of drug-likeness score for target compound **10** using Molsoft. The drug-like behavior curve is colored blue and non-drug like behavior curve is colored green.

According to Lipinski rule of five (Ro5) [35], the target compounds displayed good drug-likeness model scores and promising physicochemical properties Figure 3. The investigated compounds had one to two H-bond donors (HBD) and six H-bond acceptors (HBA). The number of rotatable bonds (nrotb) was five, indicating controlled conformational changes for good oral bioavailability. Additionally, the Log P values of the target compounds were between 1.67 and 4.10. The polar surface area (PSA) values were between 97.46 and 104.55, suggesting the good absorption and permeability of the tested compounds (Table 4). Consequentially, the newly synthesized compounds were promising drug-like candidates with Molecular Weight ≤ 500, Log P ≤ 4.15, HBA ≤ 10, HBD ≤ 5, and nrotb ≤ 10 [35,36,37].

#### 2.3.3. Density Functional Theory Study

**Figure 4:**
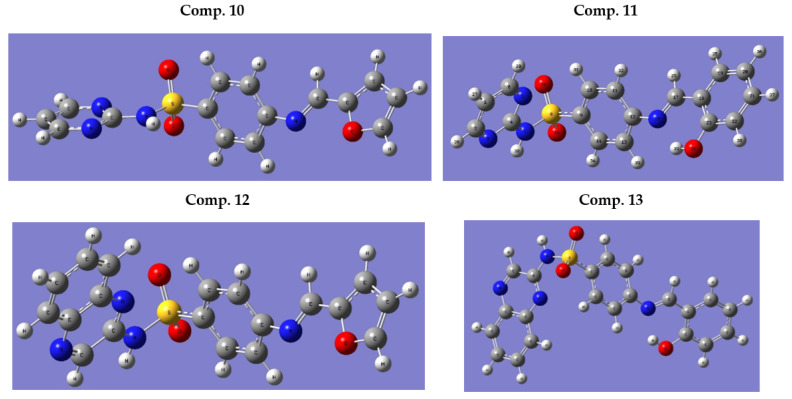
The optimized molecular structures of *E*-form for the novel compounds obtained from DFT calculation.

Structures of the tested compounds were optimized using density functional theory (DFT) in combination with Beck’s three-parameter exchange functional along with the Lee–Yang–Parr non-local correlation functional (B3LYP) [41,42,43] with the 6-31+G(d,p) basis set, which is implemented in the Gaussian 09 program package [44,45]. The quantum chemical calculation studies were performed to make a comparison between the stabilities of the newly synthesized compounds in *Z*- and *E*-forms. The quantum chemical calculations displayed that compounds **13**, **10**, **11**, and **12** were more stable in the *E*-forms than the *Z*-forms, by about 0.021, 0.053, 0.023, and 0.003 a.u., respectively, which agree with the experimental findings. Figure 4 shows the optimized molecular structures of the investigated compounds with minimum energies obtained from the calculations.

## 3. Materials and Methods

### 3.1. Chemistry

“Stuart melting point apparatus SMP10 was used for melting points determination (China). Melting points were uncorrected. Bruker Vector 22 Infrared spectrophotometer (υ_max_ in cm^−1^) (Burladingen, Germany) was used to measure infrared (IR) spectra. Nuclear Magnetic Resonance spectra were recorded on Bruker spectrometer (400 MHz) (Burladingen, Germany). Electron impact mass spectra (EI-MS) were recorded on a Finnigan MAT 312 mass spectrometer (Burladingen, Germany). Sonication was performed by Daihan (Wiseclean, D-40 kHz) sonicator (China). Microwave assisted reactions were accomplished using Milestone Startsynth lab station microwave (Via Fatebenefratelli 1/5-24010 Sorisole (BG), Italy)”.

*N*-(4-(*N*-Pyrimidin-2-ylsulfamoyl)phenyl)acetamide **4** and *N*-(4-(*N*-quinoxalin-2-ylsulfamoyl) phenyl)acetamide **5**

In a 50 mL flask, *p*-acetamidobenzenesulfonyl chloride **1** (2.34 g, 10 mmol) was added to the appropriate amine **2** or **3** (0.94 g, 10 mmol) in anhydrous pyridine (3.0 mL). A reflux condenser was connected to this system with a desiccant tube. After magnetic stirring for 4 h at 55 °C, the mixture was concentrated under vacuum for pyridine removal (bath at approximately 40–50 °C). This crude product was directly used in the following hydrolysis reaction [30].

4-Amino-*N*-(pyrimidin-2-yl)benzenesulfonamide **6** and *N*-(4-(*N*-pyrimidin-2-ylsulfamoyl) phenyl)acetamid **7**

The crude product obtained in the previous step was treated with NaOH solution (2M, 25 mL). The mixture was refluxed for 2 h at 100 °C under nitrogen. The mixture was allowed to cool to room temperature, neutralized with concentrated HCl to pH 6.0. Afterwards, the mixture was cooled in an ice bath until complete precipitation of the final product. The precipitated was filtered under vacuum, washed, and dried. The final product was re-crystallized from boiling acetonitrile/water (2:1) mixture [30].

General procedure for the synthesis of final compounds **10**–**13**.

*Method A: Conventional method*—The appropriate aldehyde **8** or **9** (0.002 moL) was added to an equimolar amount of benzene sulfonamide derivatives **6** or **7** (0.5 gm, 0.002 moL) in 10 mL 95% ethanol. The reaction mixture was stirred and refluxed until completion. The reaction mixture was poured on ice for precipitation [31]. The final product was re-crystallized from ethanol to yield the target compounds as listed in Table 5.
*Method B: Sonicated Reaction*—Benzene sulfonamide derivatives **6** or **7** (0.5 gm, 0.002 moL) and the appropriate aldehyde **8** or **9** (0.002 moL) were suspended in 5 mL of absolute ethanol. The mixture was subjected to ultrasound irradiation at 60–65 °C for suitable time until the starting material was no longer detectable via TLC. Upon completion, the reaction mixture was poured on ice [32]. The formed precipitate was filtered and re-crystallized from ethanol to yield the target compounds as listed in Table 5.
*Method C: Microwave-assisted reaction*—An equimolar mixture of benzene sulfonamide derivatives **6** or **7** (0.5 gm, 0.002 moL) and the appropriate aldehyde **8** or **9** (0.002 moL) in 5 mL 95% ethanol was irradiated by the microwave reactor at 850 watts and 100 °C until completion. The mixture was cooled and poured on ice and the separated precipitate was filtered and washed with cold absolute ethanol [33]. The resulting product was then re-crystallized from absolute ethanol to yield the target compounds as listed in Table 5.

(E)-4-(Furan-2-ylmethyleneamino)-N-(pyrimidin-2-yl)benzenesulfonamide **10**

Yellow crystals; m.p. (250–252) °C; IR (KBr, cm^−1^): 3448, 2924, 1604, 1500, 1442, 1342, 1141; EI-MS (*m*/*z*, %): 328 (M^+^); ^1^H-NMR (400 MHz) (DMSO-*d*_6_): δ_H_ 11.30 (s, 1H), 8.97 (s, 1H), 8.31–8.26 (m, 2H) 7.59–7.54 (m, 3H), 6.84–6.80 (m,1H), 6.61–6.63 (d, 2H, *J =* 8 Hz), 6.11–6.13 (d, 2H, *J =* 8 Hz); ^13^CNMR (DMSO-*d*_6_): δ_C_160.83, 158.20, 152.05, 129.61, 129.17, 113.11, 112.45. Anal.Calcd. for C_15_H_12_N_4_O_3_S:C, 54.87; H, 3.68; N, 17.06. Found: C, 55.07; H, 4.08; N, 17.46.

(*E*)-4-(2-Hydroxybenzylideneamino)-*N*-(pyrimidin-2-yl)benzenesulfonamide **11**

Yellow crystals; m.p. (251–253) °C; IR (KBr, cm^−1^): 3446, 3170, 2904, 1627, 1595, 1495, 1330, 1172; EI-MS (*m*/*z*, %): 354 (M^+^); ^1^H-NMR (400 MHz) (DMSO-*d*_6_): δ_H_12.56 (s, 1H), 11.36 (s, 1H), 8.98 (s, 1H) 8.52–8.50 (d, 2H, *J =* 8 Hz), 8.06–8.04 (dd, 1H, *J =* 8, 2 Hz),7.71–7.64 (m, 2H), 7.62–7.54 (m, 1H), 7.48–7.44 (m, 1H), 7.00–7.02 (d, 2H, *J =* 8 Hz), 6.57–6.59 (d, 2H, *J =* 8 Hz); ^13^CNMR (DMSO- *d*_6_): δ_C_161.19, 160.72, 158.73, 153.51, 129.67, 125.28, 119.97, 112.60.Anal.Calcd. for C_17_H_14_N_4_O_3_S:C, 57.62; H, 3.98; N, 15.81. Found: C, 57.22; H, 4.18; N, 16.10.

(*E*)-4-(Furan-2-*ylmethyleneamino*)-*N*-(quinoxalin-2-yl)benzenesulfonamide **12**

Light brown crystals; m.p. (255–257) °C; IR (KBr, cm^−1^): 3425, 2924, 1650, 1534, 1481, 1388, 1110; EI-MS (*m*/*z*, %): 378 (M^+^); ^1^H-NMR (400 MHz) (DMSO-*d*_6_): δ_H_ 11.68 (s, 1H), 8.98 (s, 1H), 8.58 (s, 1H) 7.93–7.80 (m, 2H), 7.78–7.71 (m,3H), 7.62–7.58 (m, 2H), 6.61–6.59 (d, 2H, *J =* 8 Hz), 6.19–6.21 (d, 2H, *J =* 8 Hz); ^13^CNMR (DMSO-*d*_6_): δ_C_153.80, 146.90, 139.18, 138.35, 131.15, 130.55, 129.15, 127.90, 127.54. 112.69.Anal.Calcd. for C_19_H_14_N_4_O_3_S:C, 60.31; H, 3.73; N, 14.81. Found: C, 60.60; H, 4.12; N, 15.14.

(*E*)-4-(2-*Hydroxybenzylideneamino*)-*N*-(quinoxalin-2-yl)benzenesulfonamide **13**

Light brown crystals; m.p. (254–256) °C; IR (KBr, cm^−1^): 3425, 3147, 3047, 1600, 1551, 1450, 1373, 1195; EI-MS (*m*/*z*, %): 404 (M^+^); ^1^H-NMR (400 MHz) (DMSO *d*_6_): δ_H_ 12.50 (s, 1H), 11.58 (s, 1H), 8.96 (s, 1H) 8.58 (s, 1H),8.20–8.18 (m,2H), 7.96–7.92 (m, 2H), 7.81–7.72 (m, 1H), 7.69–7.59 (m, 2H), 7.57–7.43 (m, 1H), 7.01–6.99 (d, 2H, *J =* 8 Hz), 6.60–6.58 (d, 2H, *J =* 8 Hz); ^13^CNMR (DMSO-*d*_6_): δ_C_161.20, 153.87, 146.73, 136.92, 131.20, 130.58, 129.18, 127.65, 119.96, 117.69, 112.70.Anal.Calcd. for C_21_H_16_N_4_O_3_S:C, 62.36; H, 3.99; N, 13.85. Found: C, 62.00; H, 4.19; N, 14.25.

### 3.2. Biological Evaluation

The experimental protocols were approved by the Ethics Committee of the Suez Canal University with code 202403R1.

#### 3.2.1. Cyclooxygenase Inhibition Assays

The ability of the test compounds to inhibit human recombinant COX-1 and human recombinant COX-2 (IC_50_ value, µM) was assigned using an enzyme immune assay (EIA) kit (item no. 560131, Cayman Chemical, Ann Arbor, MI, USA) according to the previously reported method [46].

#### 3.2.2. The Anti-Platelet Aggregation Assays

The anti-platelet aggregation assays were performed according to the method reported by XIE et al. [47] (Supplementary Materials).

### 3.3. In Silico Studies

#### 3.3.1. Molecular Docking

The X-ray crystal structures of two proteins with the co-crystallized ligands crystal structure of cyclooxygenase-1 in complex with celecoxib (PDB 3KK6) [48] and crystal structure of cyclooxygenase-2 in complex with celecoxib (PDB 3LN1) [49] were downloaded from the Protein Data Bank. The in silico studies were carried out using AutoDock Vina software [50] with Chimera-UCSF [51]. Chimera was used to tantalize both the interactive analysis as well as binding disposition.

#### 3.3.2. Bioinformatics Study

Bioinformatics study of the novel compounds were calculated using Molinspiration [38], SwissADME [40] website and MolSoft web-based software [39].

#### 3.3.3. Density Functional Theory Study

The DFT study was performed according to the method reported by Awad et al. in 2018 [52].

## 4. Conclusions

Four new Schiff base analogues **10**–**13** were synthesized using the conventional method, sonication, and microwave-assisted methods. Biological results revealed that all the screened compounds hold promise as potential anti-platelet aggregation agents. They displayed IC_50_ values ranging from 0.11 to 0.37 μM against ADP and IC_50_ values ranging from 0.12 to 1.03 μM against collagen. These ranges for compounds **10**–**13** were superior to those of the standard aspirin with IC_50_ = 0.49 μM against ADP, but only compound **12** (IC_50_ = 0.12 μM) was found more potent than the standard aspirin with IC_50_ = 0.51 μM against collagen. Additionally, they exhibited notable COX-1-inhibitory activity, with IC_50_ values ranging from 0.71 μM to 4.82 μM and COX-1 selectivity indices ranging from 2.87 to 18.69. These findings suggest that compounds **10**–**13** hold potential as dual-function agents with anti-platelet aggregation and COX-1-inhibitory properties, warranting further investigation into their therapeutic applications. In silico molecular modeling was performed for all the target compounds inside the platelet COX-1 active site, and their binding interactions were not hindered by celecoxib. The geometric molecular structures were deduced through computational chemistry calculations with the B3LYP/6-31+G (d,p) level to analyze the stable forms of the novel compounds. The calculations showed that E-forms are more stable than *Z*-form for the investigated compounds, which are experimentally in a good correlation with the NOESY.

## Figures and Tables

**Table 1:** The cyclooxygenase-inhibitory activities of new compounds **10**–**13**.

Comp.	IC_50_ (μM)		COX1 Selectivity Index
	COX-1	COX-2	
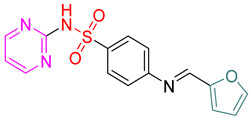 **10**	1.92	11.26	5.86
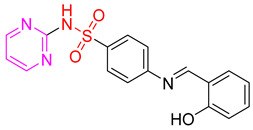 **11**	4.82	15.24	3.16
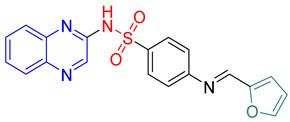 **12**	0.71	13.27	18.69
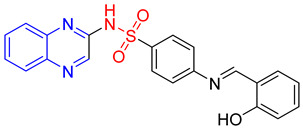 **13**	3.22	9.26	2.87
Celecoxib	14.20	0.42	0.02
Indomethacin [34]	0.26	2.63	10.11
Benzene sulfonamide derivative (I) [13]	12	˃100	≤100
Stilbene derivative (II) [13]	15	˃200	≤200

**Table 2:** Anti-platelet aggregation assay of the tested compounds.

Comp.	ADP		Collagen	
	Inhibition Rate (%) ± SD *	IC_50_ (μM)	Inhibition Rate (%) ± SD *	IC_50_ (μM)
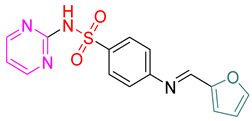 **10**	78.36 ± 1.36	0.37	89.36 ± 1.23	0.62
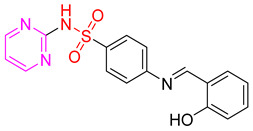 **11**	84.36 ± 1.64	0.25	76.35 ± 1.34	1.03
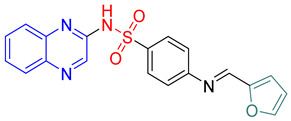 **12**	98.1 ± 1.68	0.11	95.9 ± 1.39	0.12
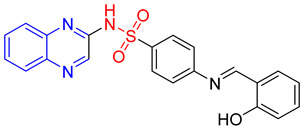 **13**	46.32 ± 1.32	NA	45.36 ± 1.56	NA
Aspirin	92.36 ± 1.5	0.49	64.3 ± 1.38	0.51

* Values are expressed as means ± SDs of three independent values. Values were calculated through non-linear regression curve fit of 4 working concentrations. NA = not active.

**Table 3:** Binding energies and binding interactions of the target compounds in COX-1 and COX-2.

Comp.	COX-1		COX-2	
	Binding Energy (Kcal/mol)	Binding Interactions	Binding Energy (Kcal/mol)	Binding Interactions
Celecoxib	−16.91	4 HB with Gln 192, His 90, Ser 353 and Ser 516	−14.45	3 HB with Leu 338, Gln 178, and Ser 339
**10**	−16.44		−9.87	1 HB with Ser 516
**11**	−17.25		−8.94	Arg 499 and His 75
**12**	−20.70		−5.89	1 HB and Arene-cation with Arg 4991 HB with His 75
**13**	−19.98		−8.84	2 HB with Arg 106 and Gln 178

**Table 4:** In silico ADME pharmacokinetic properties of the target compounds **10**–**13**.

Comp.	Molinspiration 2018.10 [38]						MolSoft [39]			SwissADME [40]
	MWt (D)	MV (A^3^)	PSA (A^2^)	Log P	Nrotb	Nviolations	HBA	HBD	Solubility (mg/L)	Drug-Likeness (Lipinski–Pfizer Filter)
**10**	328.35	267.21	97.46	1.67	5	0	6	1	683.14	“Yes, drug like” MW ≤ 500, Log P ≤ 4.15, HBA ≤ 10 and HBD ≤ 5
**11**	354.39	293.66	104.55	2.35	5	0	6	2	574.55	
**12**	378.41	311.20	97.46	3.41	5	0	6	1	102.89	
**13**	404.45	337.65	104.55	4.10	5	0	6	2	116.32	

MWt: Molecular Weight, MV: Molecular Volume, PSA: Polar Surface Area, Log P: Octanol-water partition coefficient, nrotb: number of rotatable bonds, nviolations: number of violations, HBA: Hydrogen Bond Acceptor, HBD: Hydrogen Bond Donor.

**Table 5:** The reaction times and yields of target compounds **10**–**13**.

Comp.	Method	Reaction Time	Yield%
**10**	Method A: Conventional method	2 h	75%
	Method B: Sonicated reaction	10 min	80%
	Method C: Microwave-assisted reaction	5.5 min	89%
**11**	Method A: Conventional method	2 h	74%
	Method B: Sonicated reaction	10 min	83%
	Method C: Microwave-assisted reaction	5.5 min	88%
**12**	Method A: Conventional method	3 h	77%
	Method B: Sonicated reaction	15 min	85%
	Method C: Microwave-assisted reaction	8.5 min	92%
**13**	Method A: Conventional method	3 h	75%
	Method B: Sonicated reaction	15 min	84%
	Method C: Microwave-assisted reaction	8.5 min	90%

## Data Availability

Data are contained within the article.

## Supplementary Materials

The following supporting information can be downloaded at: https://www.mdpi.com/article/10.3390/ph17060710/s1, NMR spectrum and the anti-platelet aggregation assays.
