# Supplementary material for: Synthesis, Docking, and DFT Studies on Novel Schiff Base Sulfonamide Analogues as Selective COX-1 Inhibitors with Anti-Platelet Aggregation Activity
Source: Pharmaceuticals (Basel). 2024 May 30;17(6):710. doi: 10.3390/ph17060710 (PMC11206759; doi:10.3390/ph17060710)
Supplement: Supplementary file 1 [file pharmaceuticals-17-00710-s001.zip › pharmaceuticals-3006882-supplementary.pdf]

SUPPORTING INFORMATION

Synthesis, Docking, and DFT Studies on Novel Schiff Base Sulfonamide Analogues as Selective COX-1 Inhibitors with Anti-Platelet Aggregation Activity

Yasmine M. Abdel Aziz [1,*,†], Mohamed S. Nafie [2,3,†], Pierre A. Hanna [4], Sherif Ramadan [5], Assem Barakat [6,*] and Marwa Elewa [1]

[1] Pharmaceutical Organic Chemistry Department, Faculty of Pharmacy, Suez Canal University, Ismailia 41522, Egypt; marwa_elewa@pharm.suez.edu.eg
[2] Department of Chemistry, College of Sciences, University of Sharjah, Sharjah P.O. Box 27272, United Arab Emirates; mohamed.elsayed@sharjah.ac.ae or mohamed_nafie@science.suez.edu.eg
[3] Chemistry Department, Faculty of Science, Suez Canal University, Ismailia P.O. Box 41522, Egypt
[4] Department of Pharmaceutics and Industrial Pharmacy, Faculty of Pharmacy, Suez Canal University, Ismailia 41522, Egypt; pierre_hanna@pharm.suez.edu.eg
[5] Chemistry Department, Michigan State University, East Lansing, MI 48824, USA; sramadan@chemistry.msu.edu
[6] Department of Chemistry, College of Science, King Saud University, P.O. Box 2455, Riyadh 11451, Saudi Arabia
* Correspondence: yasmine_abdelaziz@pharm.suez.edu.eg (Y.M.A.A.); ambarakat@ksu.edu.sa (A.B.)
† These authors contributed equally to this work.

^{1}H-NMR (DMSO- d_6) of compound 10

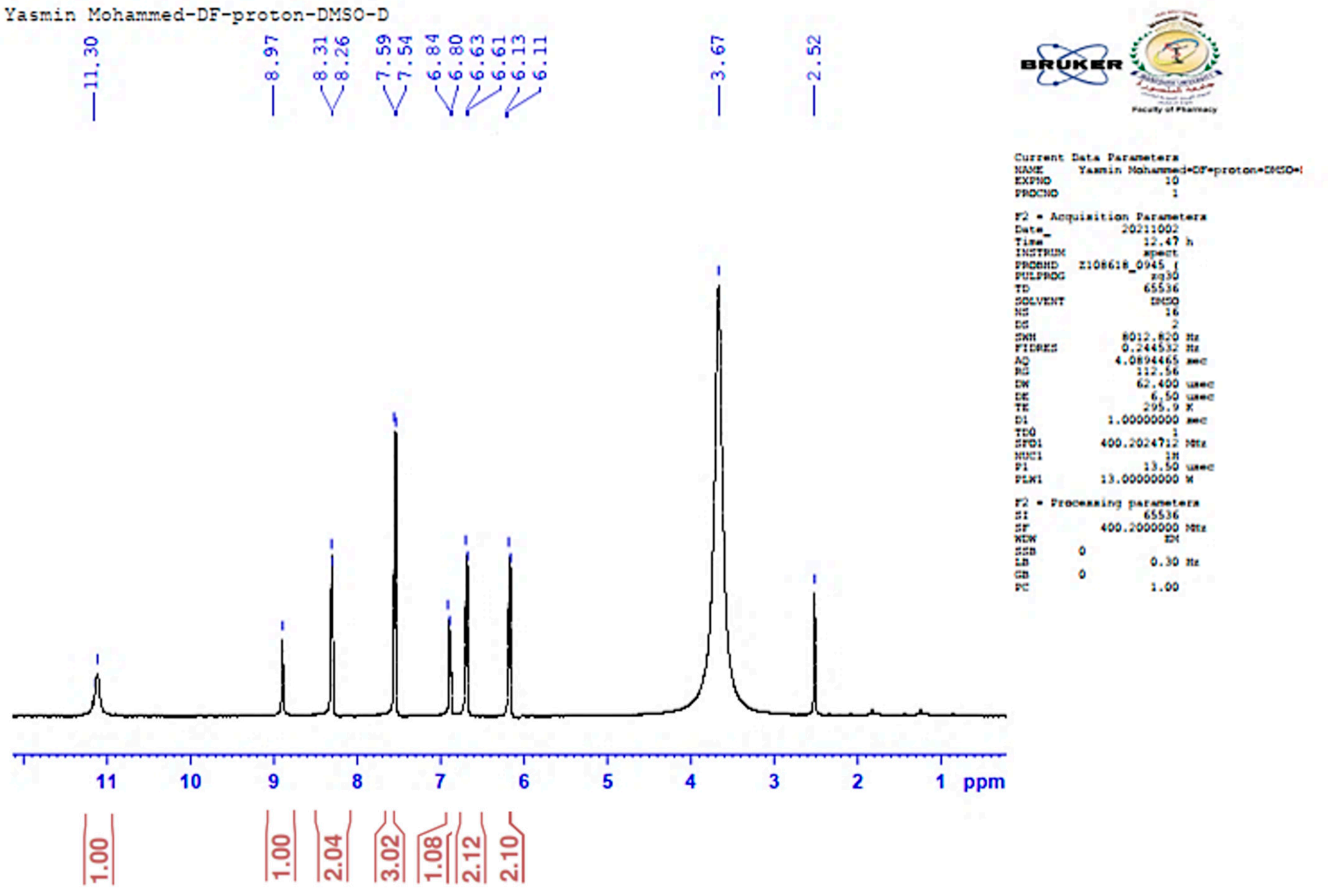

^{13}C-NMR (DMSO-d_6) of compound 10

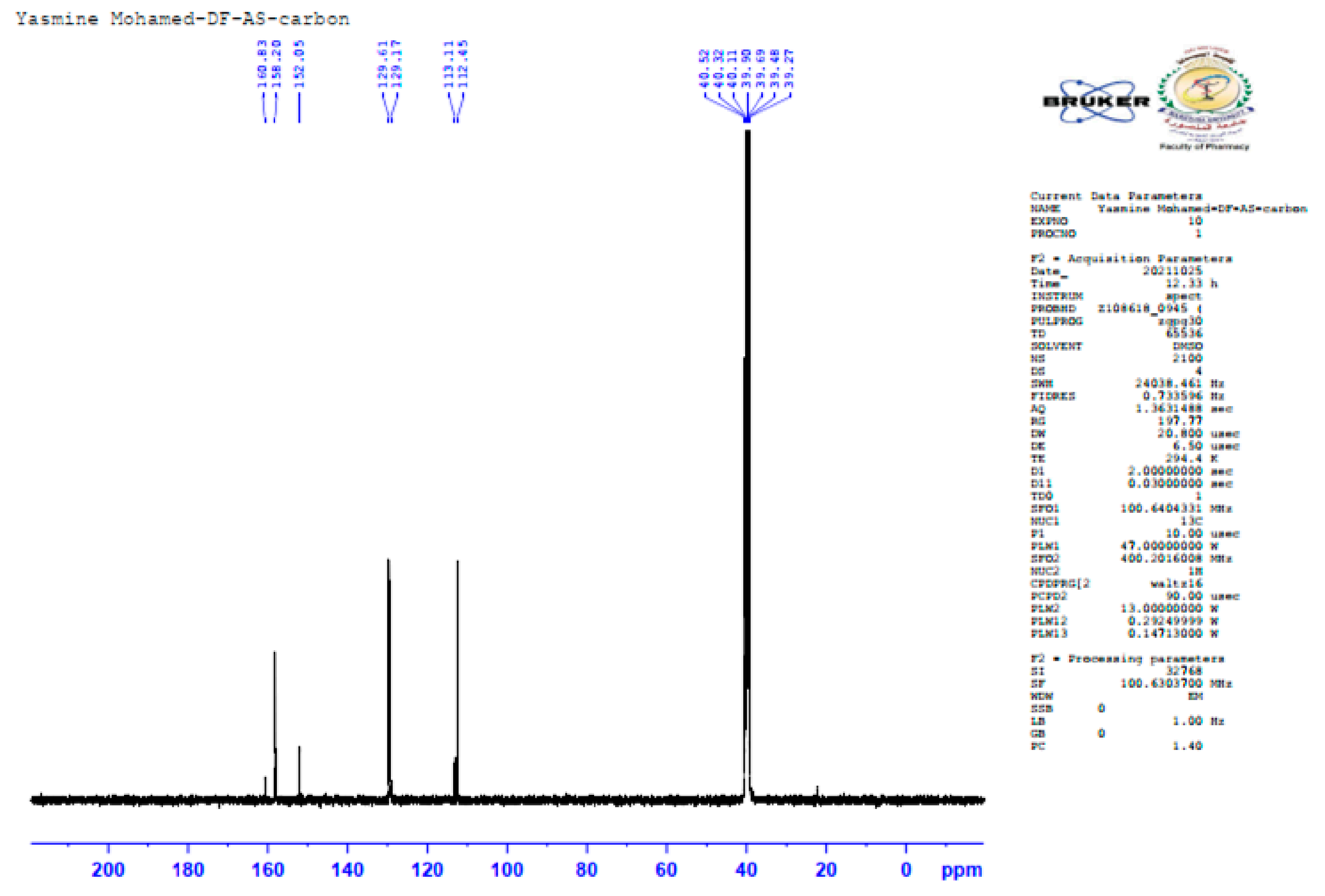

NOESY of compound 10

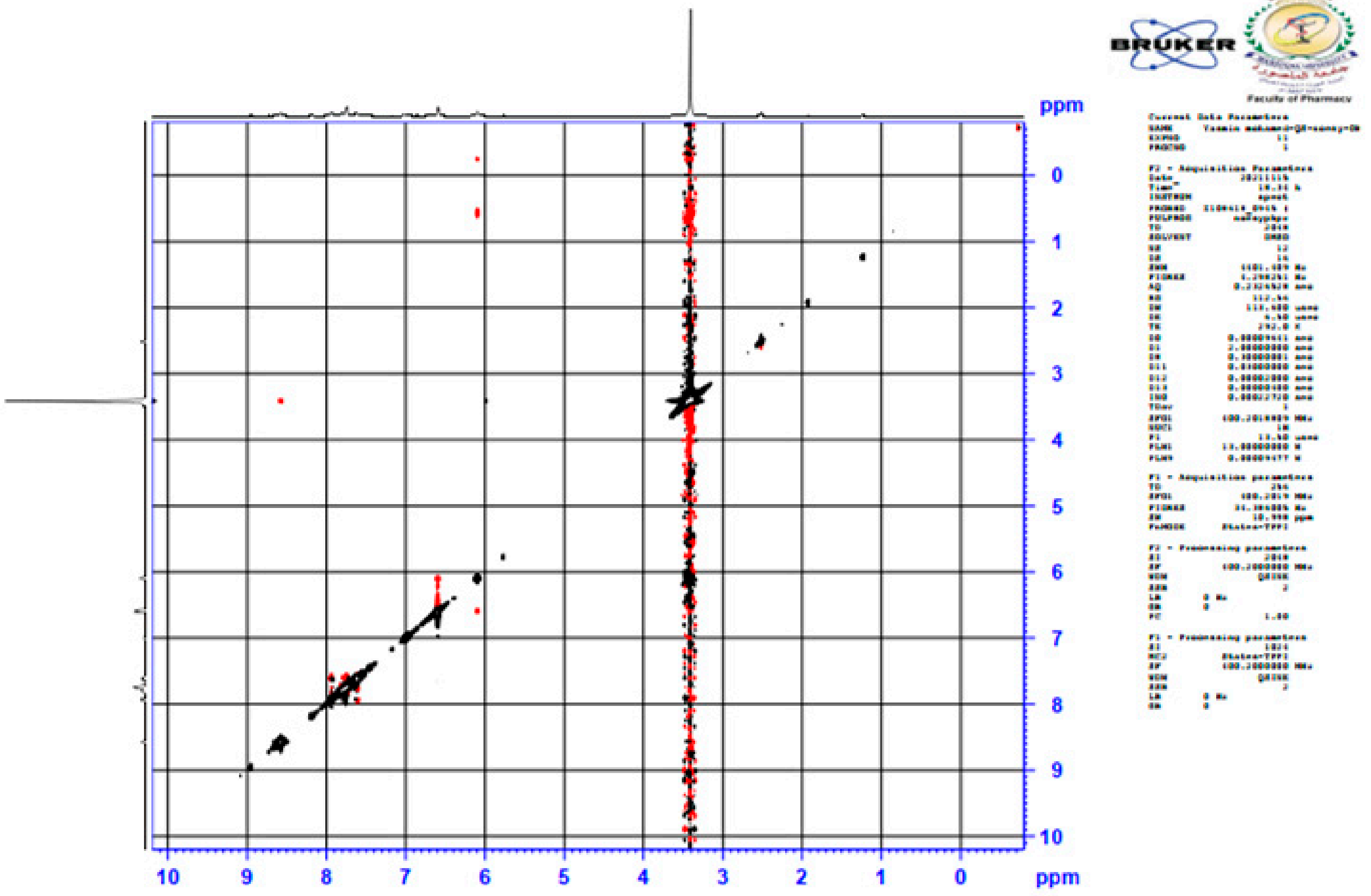

EI-MS (m/z, %)of compound 10

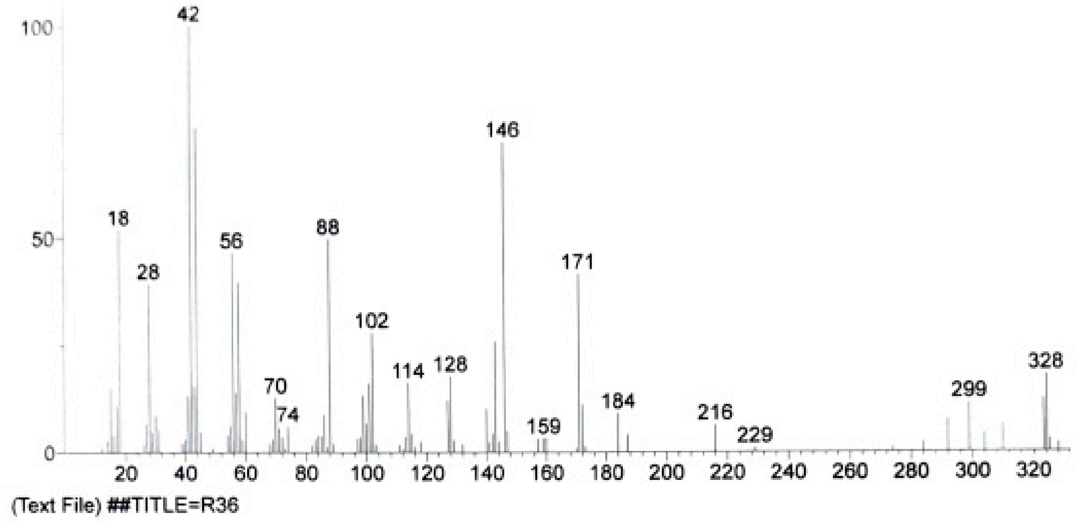

IR (KBr, cm^{-1}) of compound 10

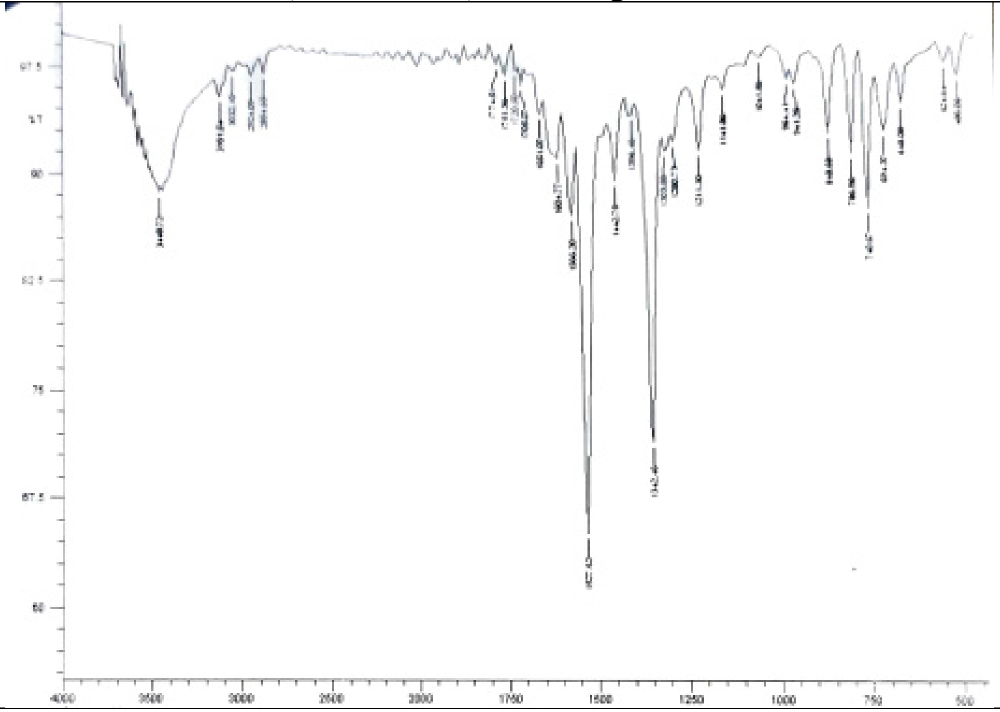

^{1}H-NMR (DMSO-d_6) of compound 11

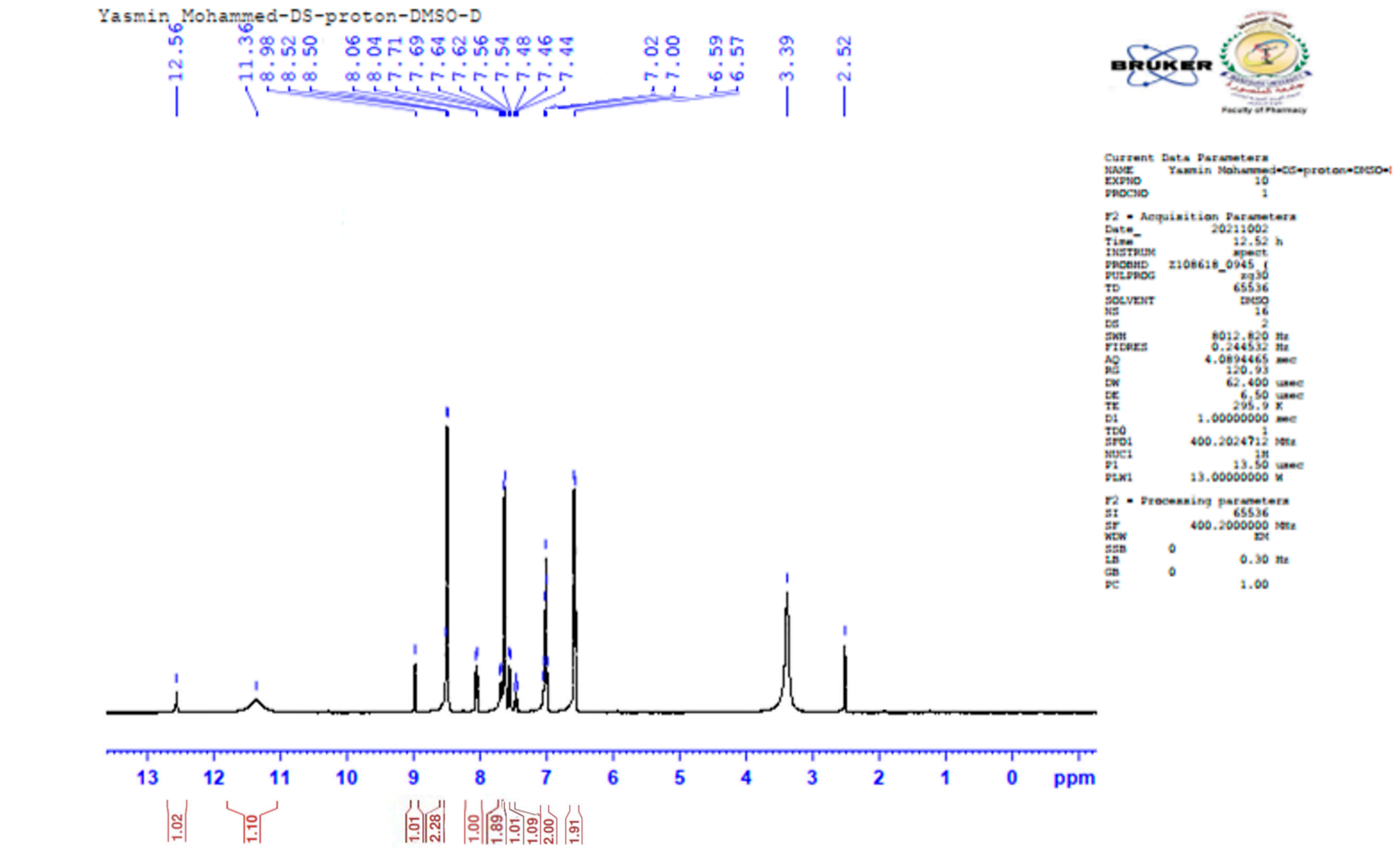

^{13}C-NMR (DMSO-d_6) of compound 11

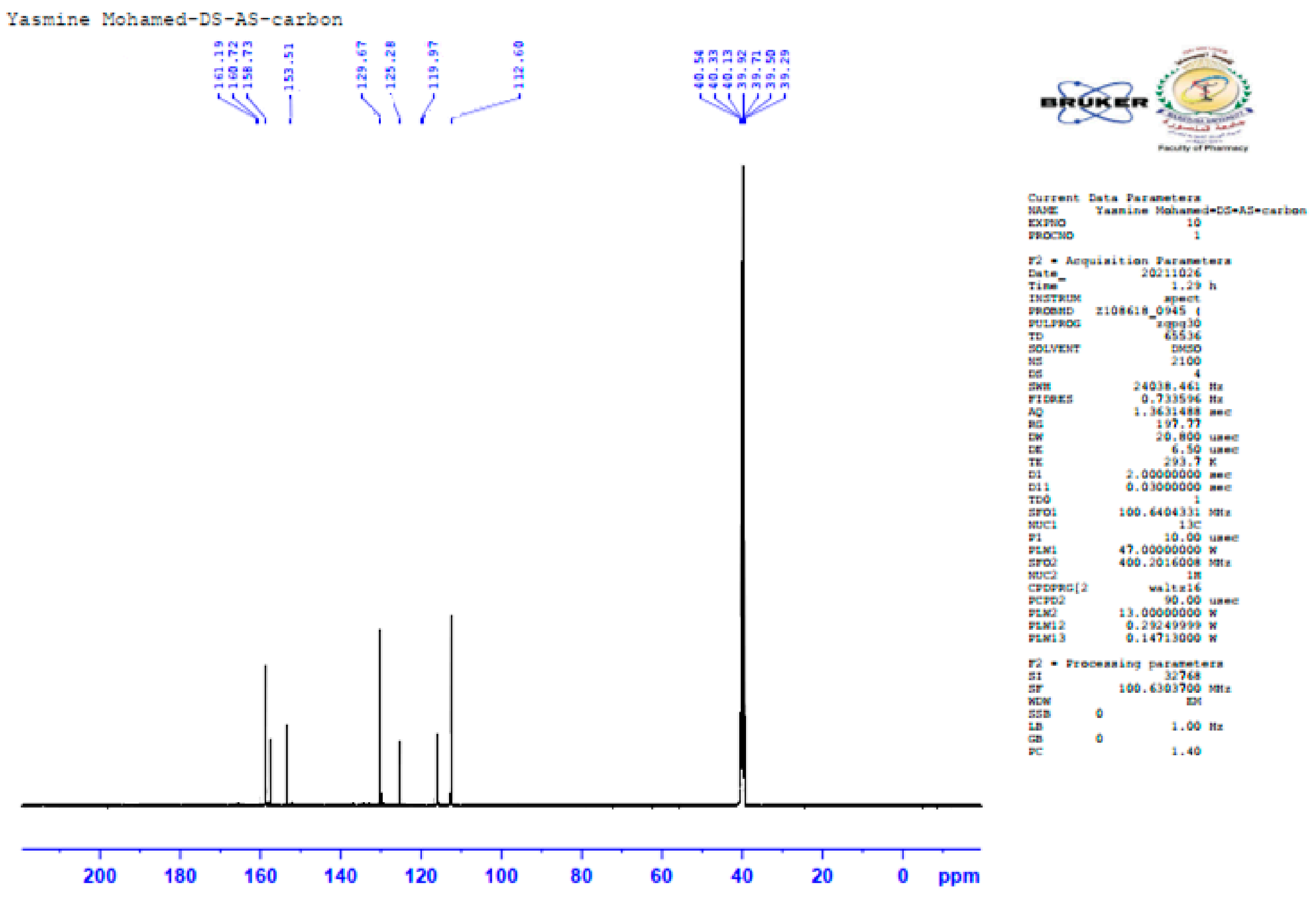

NOESY of compound 11

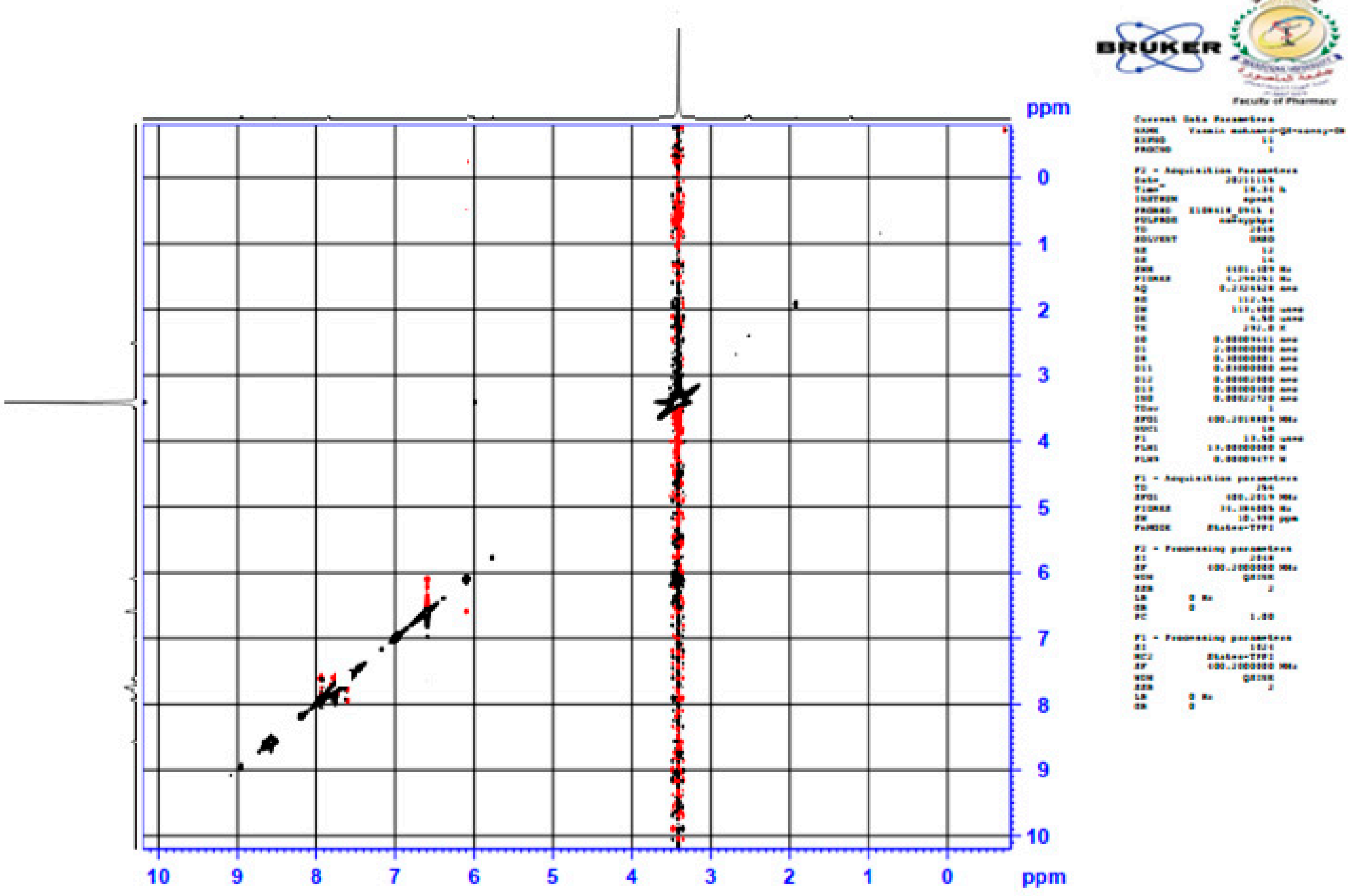

EI-MS (m/z, %)of compound 11

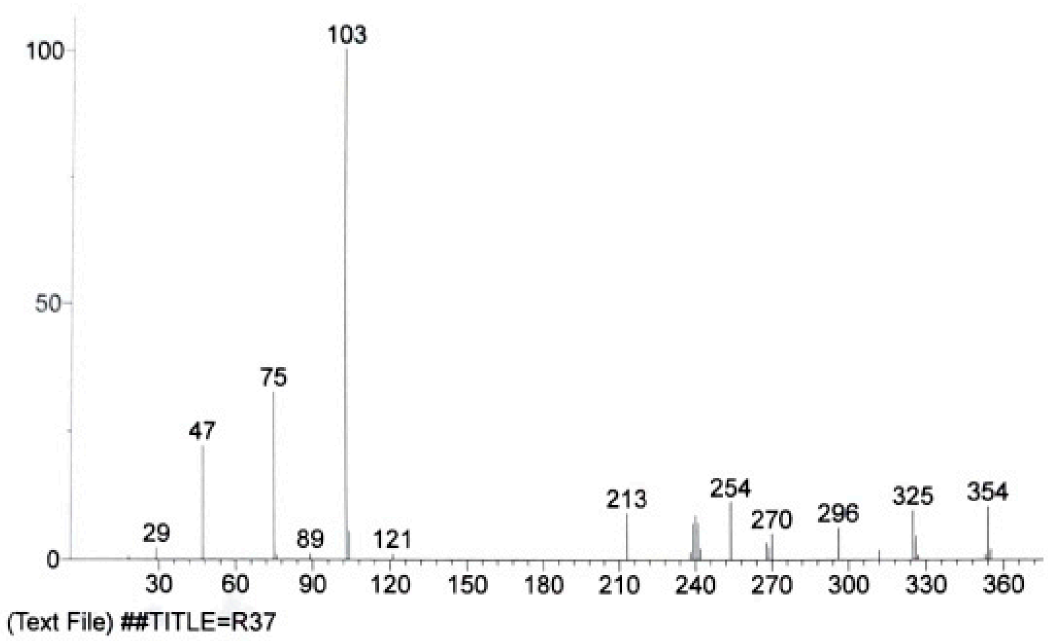

IR (KBr, cm^{-1}) of compound 11

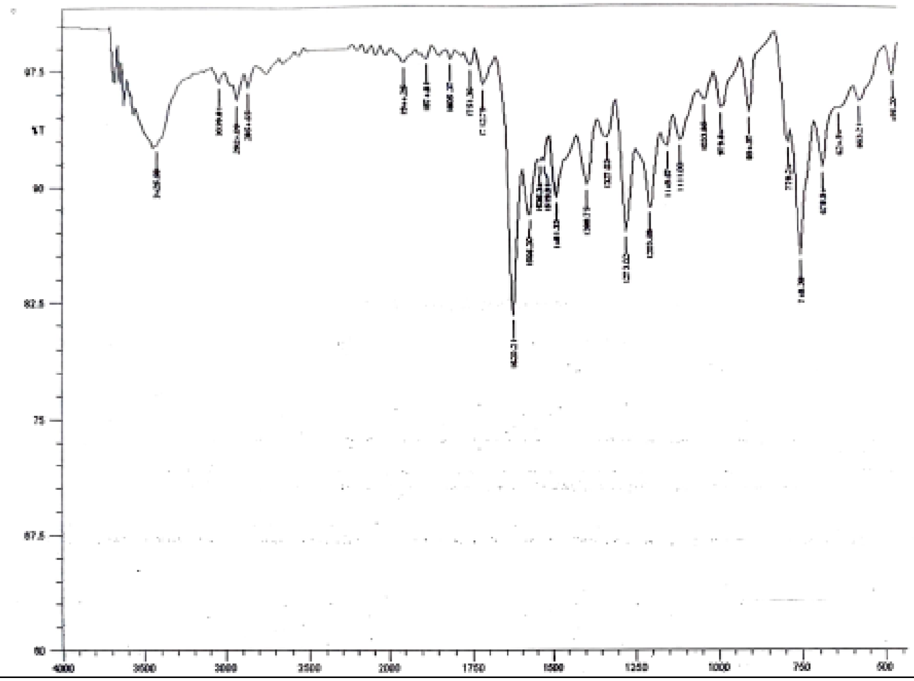

^{1}H-NMR (DMSO-d_6) of compound 12

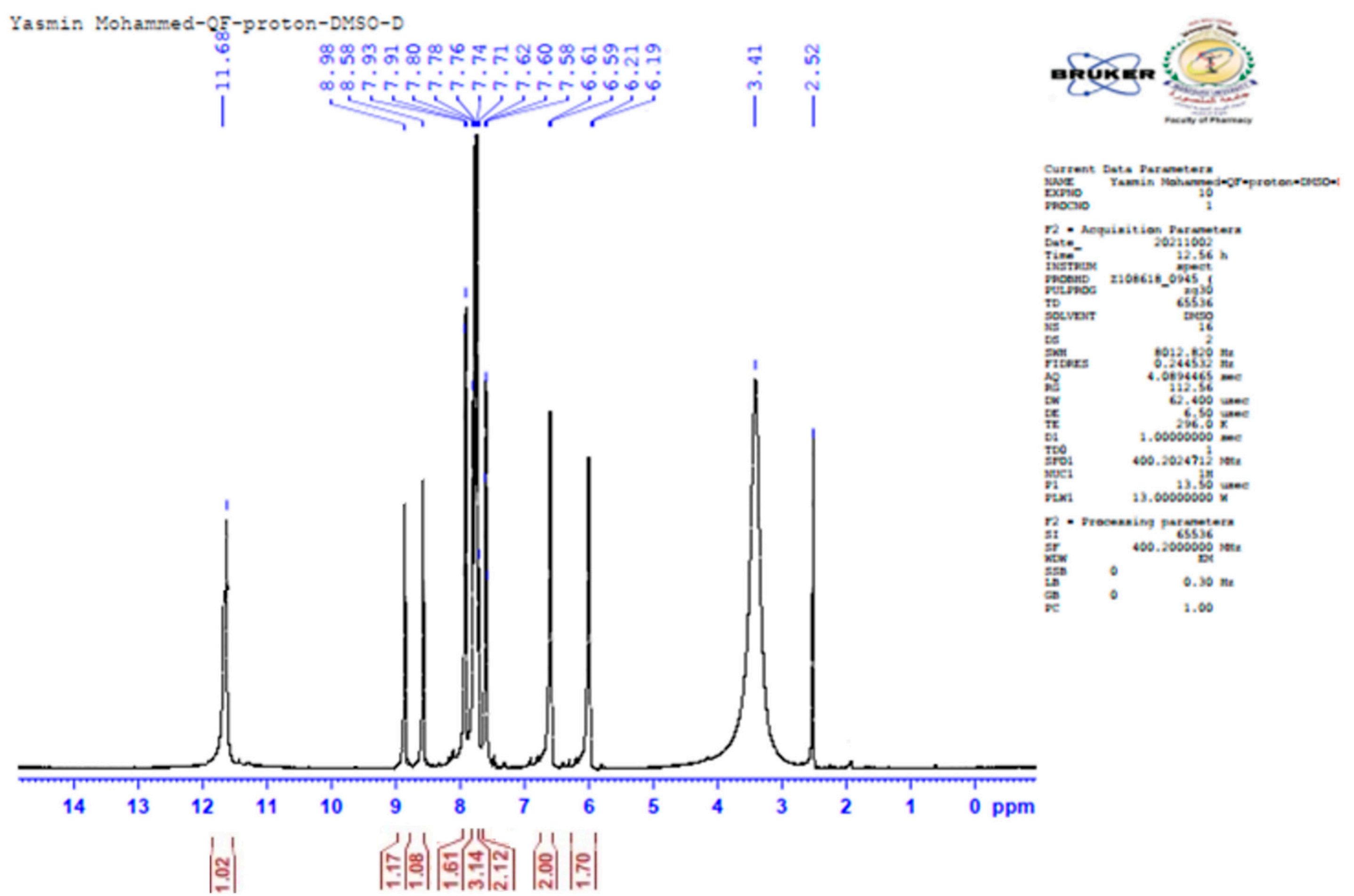

^{13}C-NMR (DMSO-d_6) of compound 12

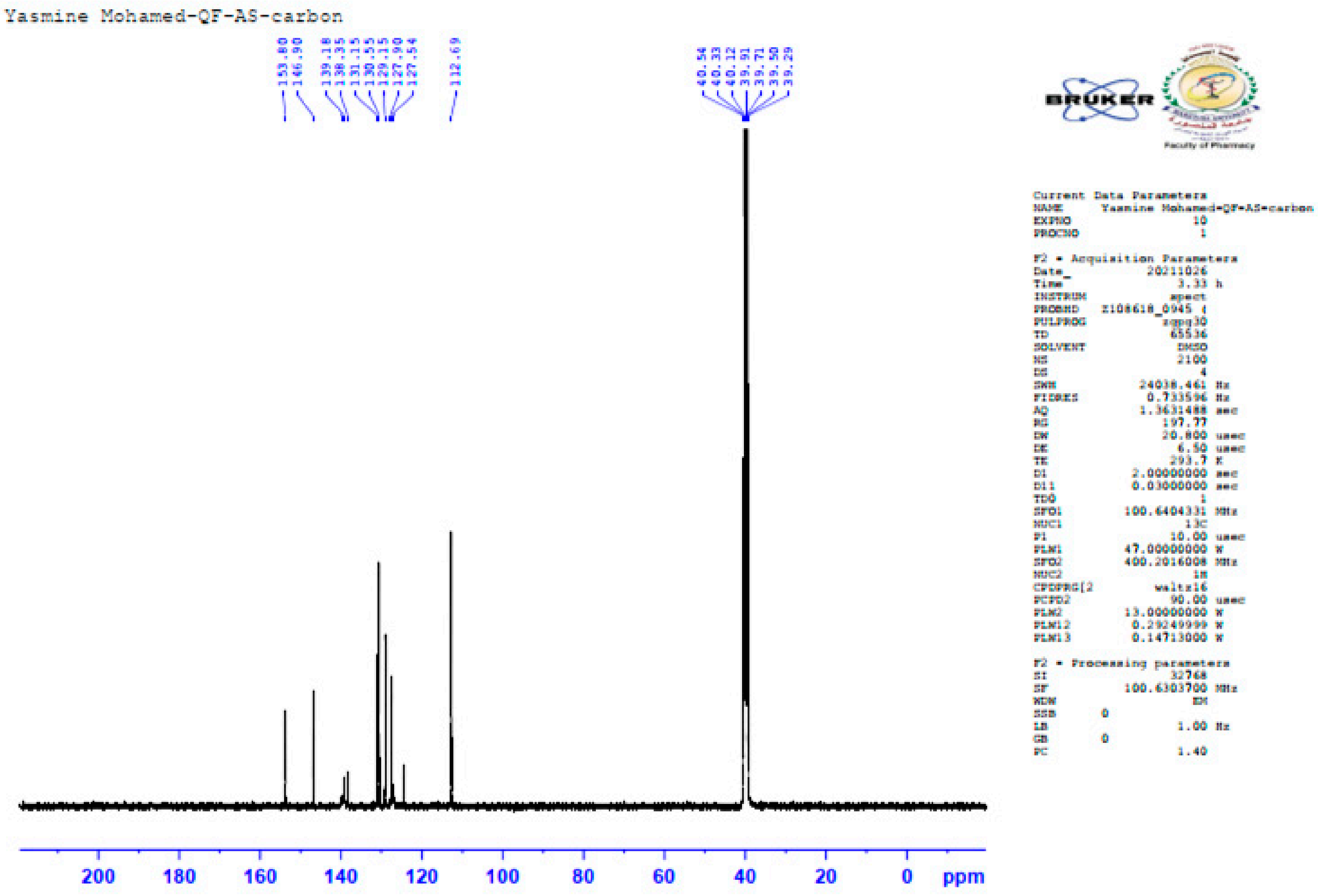

NOESY of compound 12

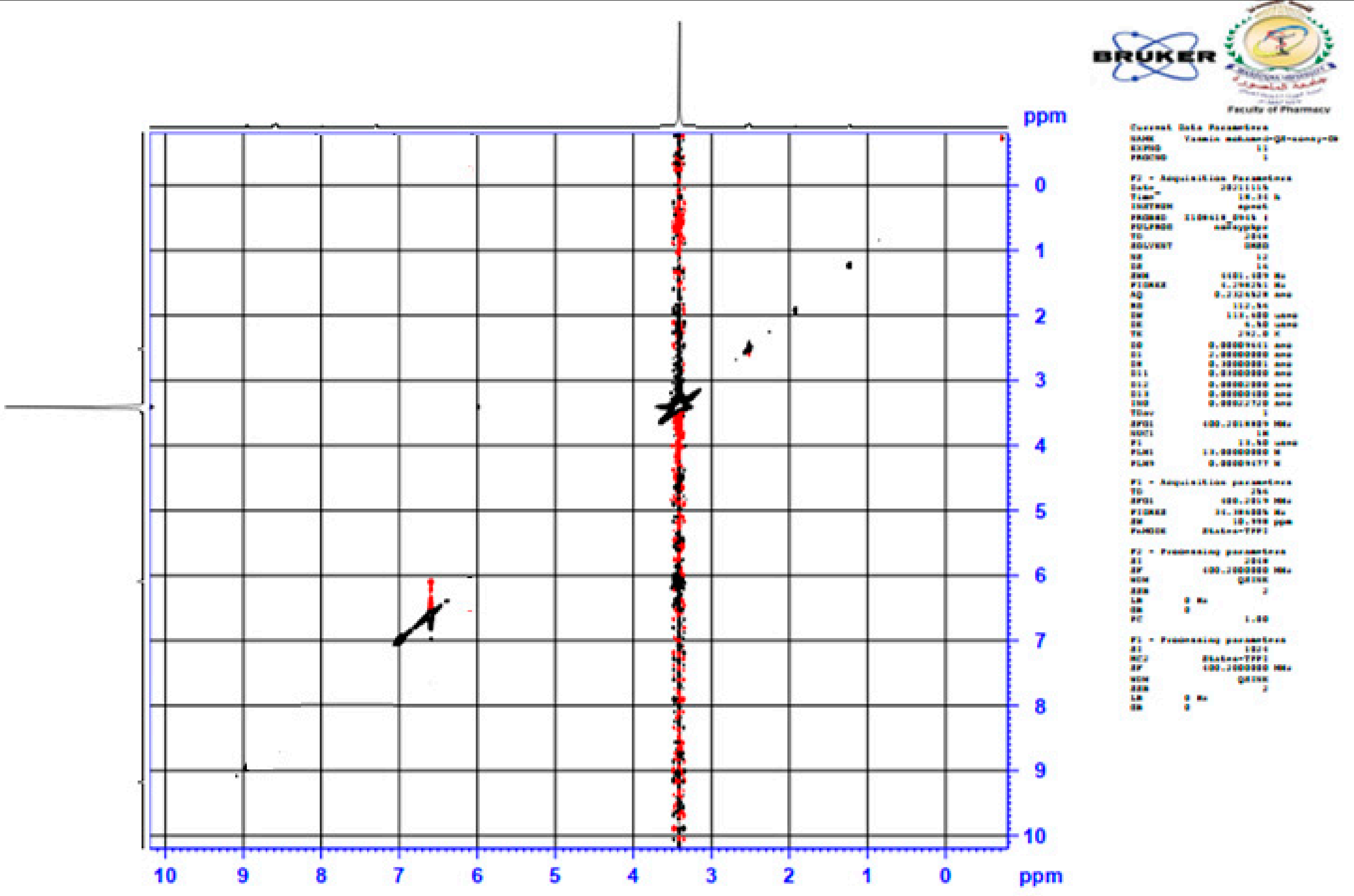

EI-MS (m/z, %)of compound 12

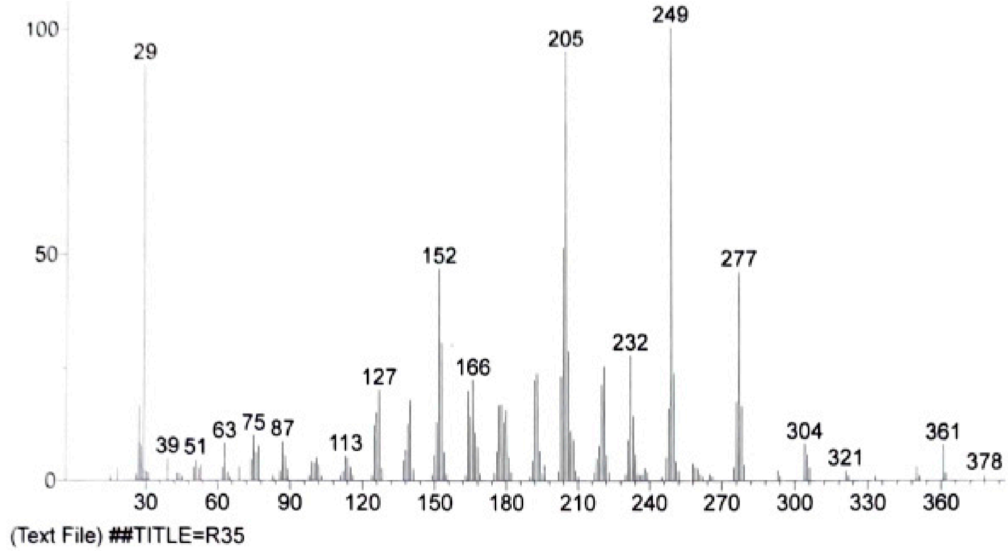

IR (KBr, cm^{-1}) of compound 12

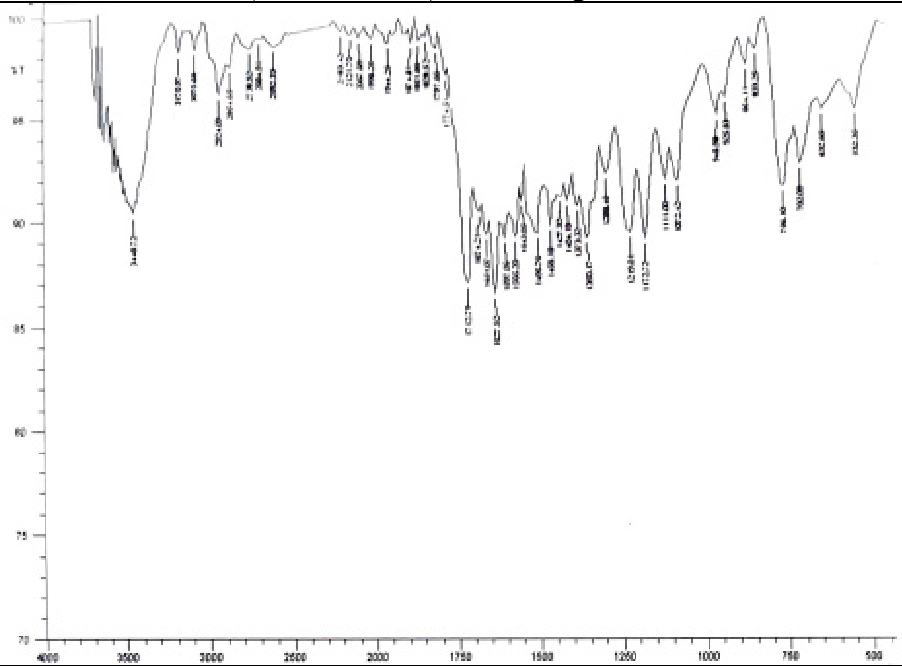

^{1}H-NMR (DMSO-d_6) of compound 13

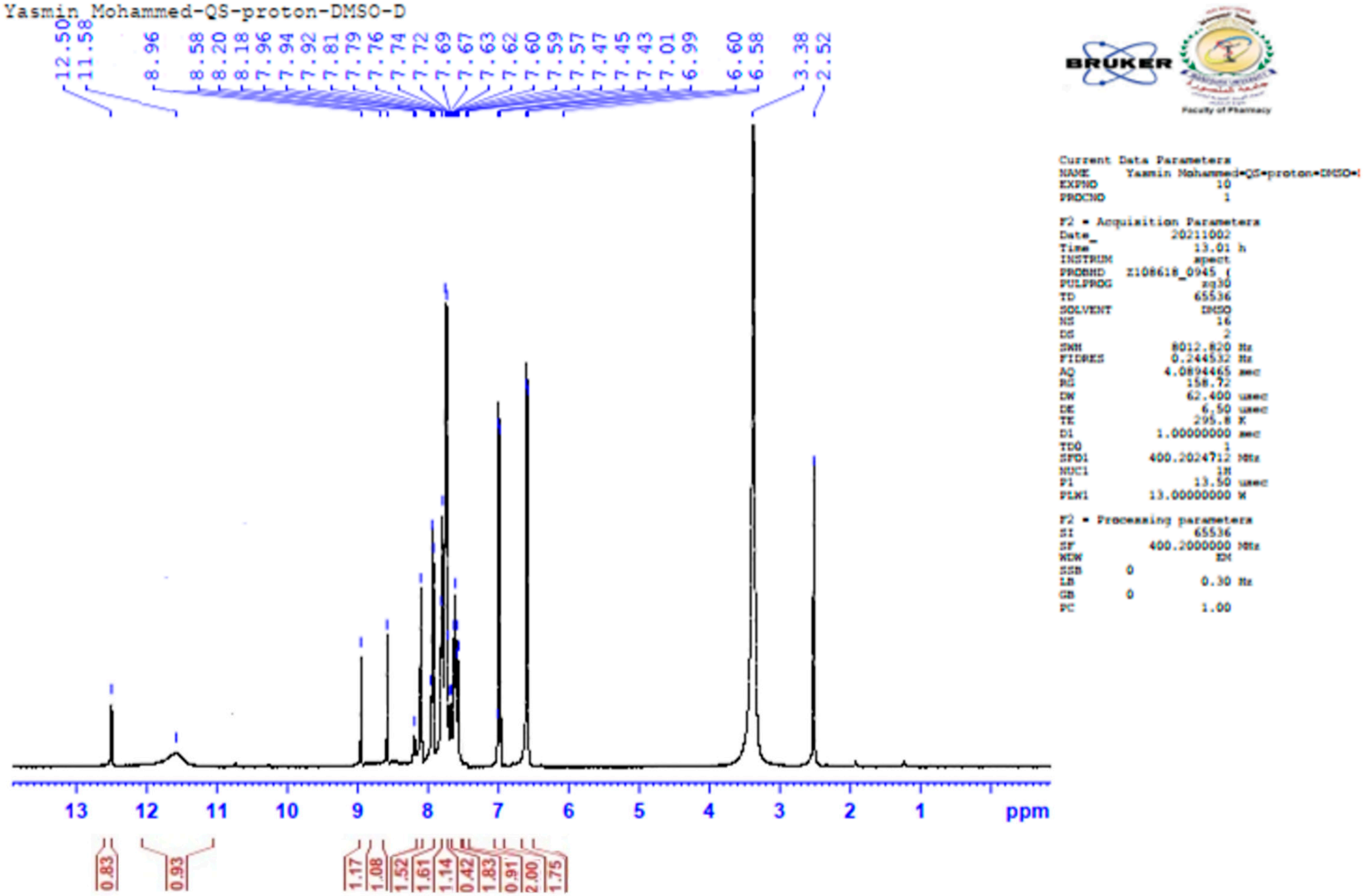

^{13}C-NMR (DMSO-d_6) of compound 13

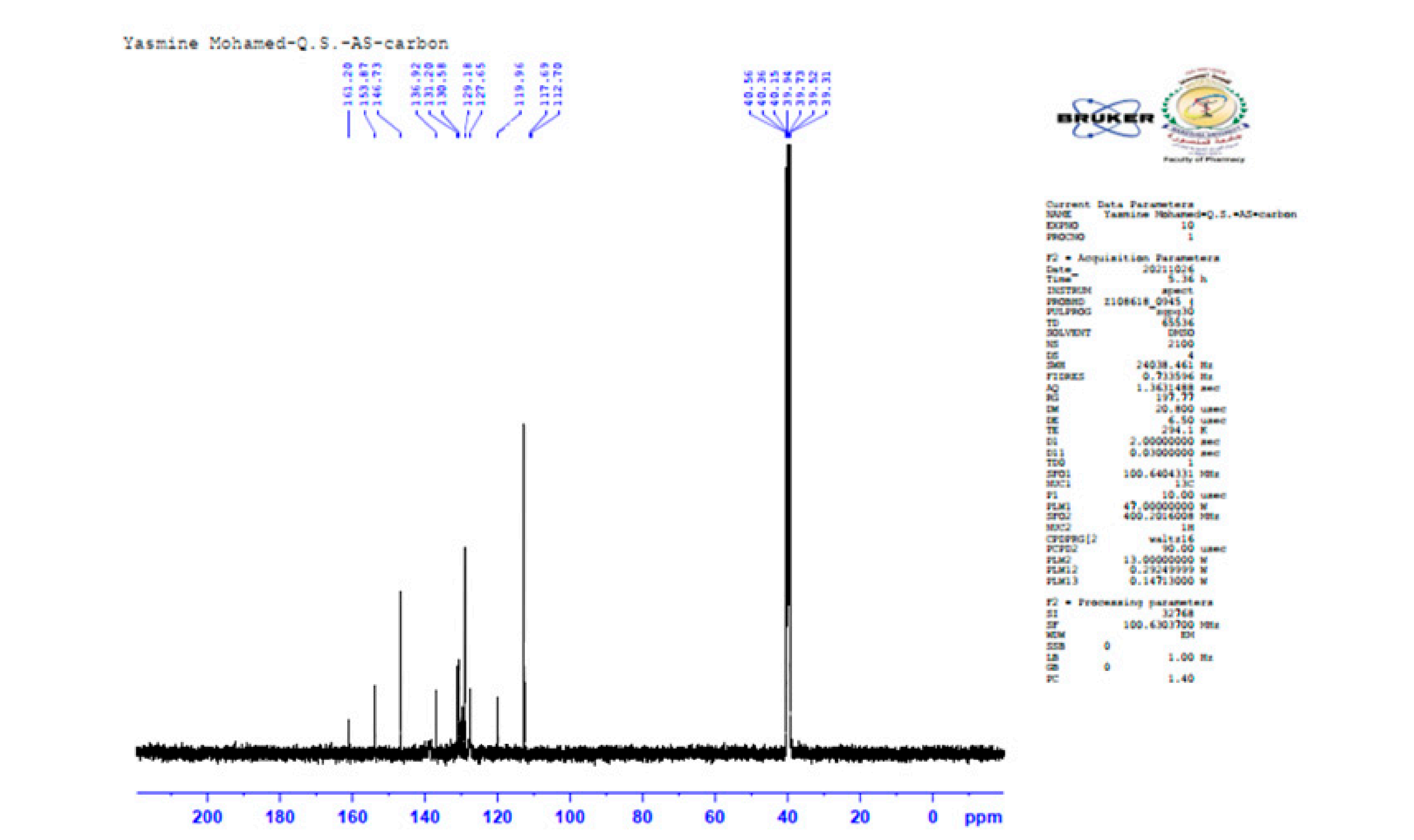

NOESY of compound 13

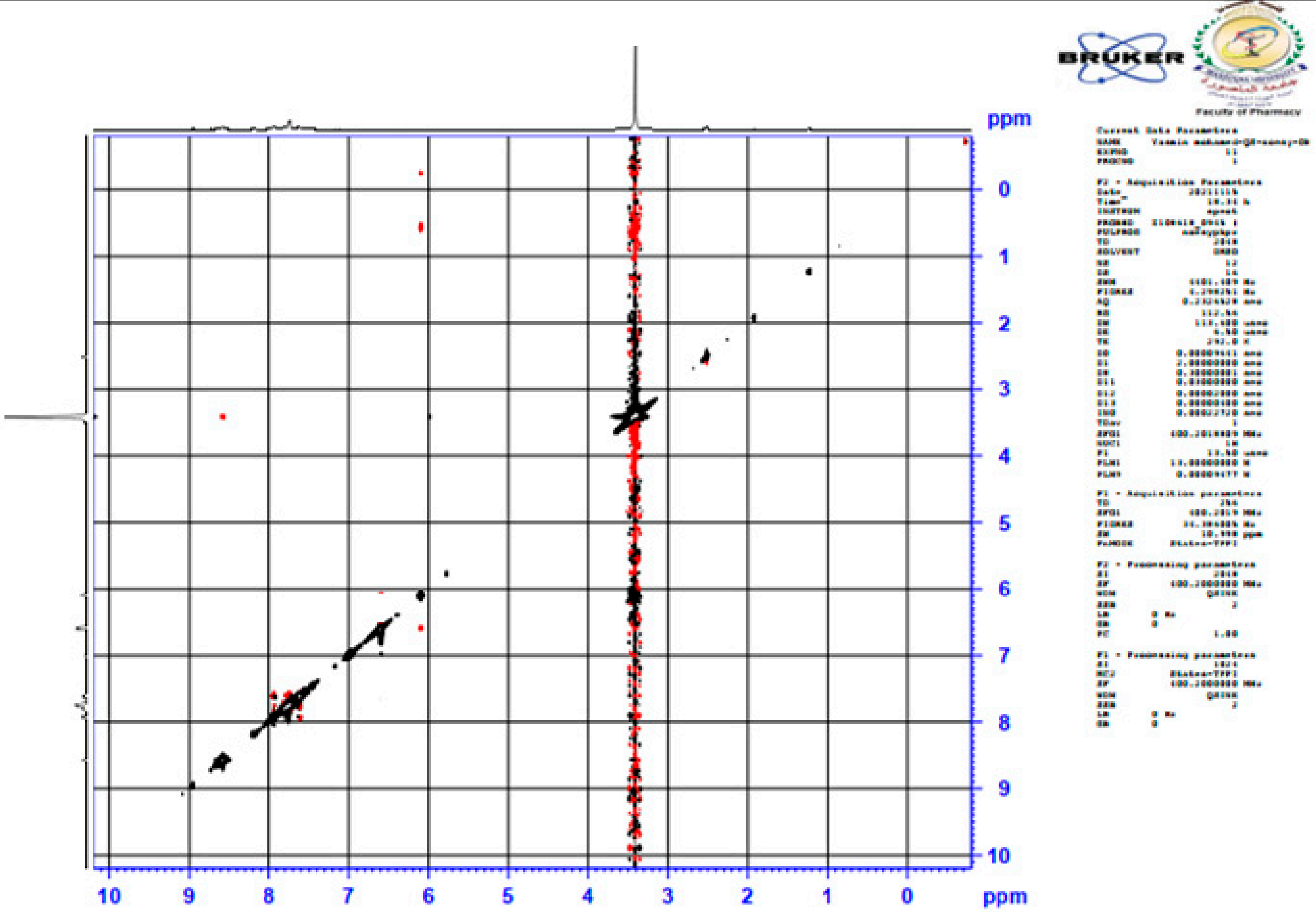

EI-MS (m/z, %)of compound 13
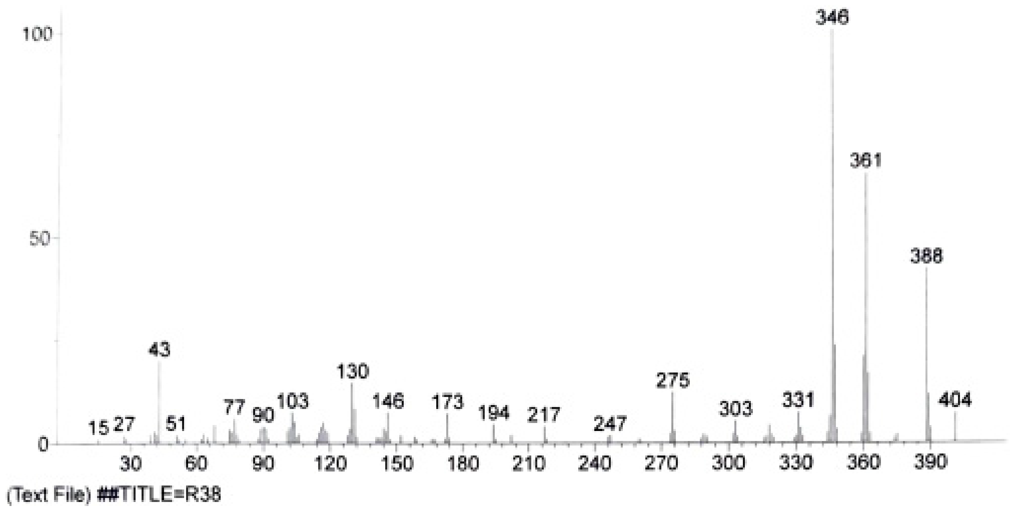

IR (KBr, cm^{-1}) of compound 13

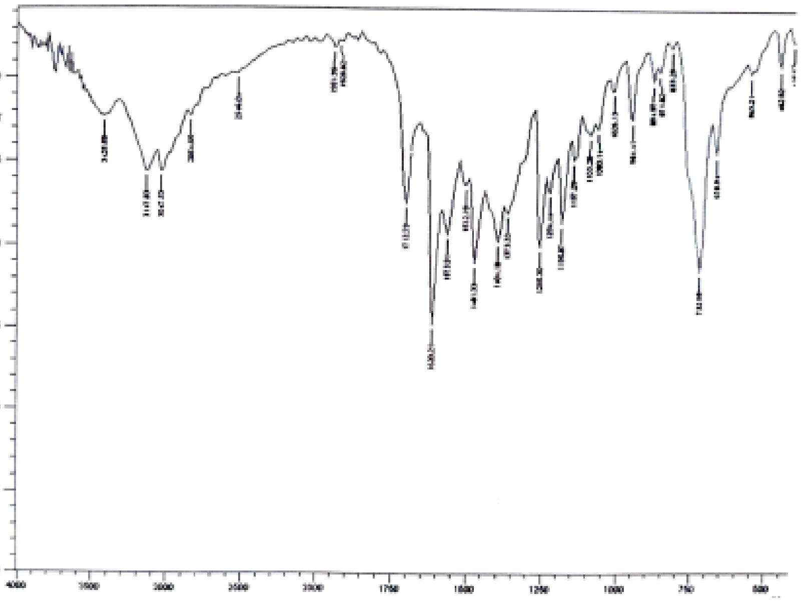

The anti-platelet aggregation Assays

Platelet aggregation was measured at 37°C in a platelet aggregometer (PRECILBY-NJ4, Pu Lisheng Corp. Beijing, PR China). Platelet-poor plasma was prepared by centrifugation at 3000× g for 10 min from PRP, removed with a pipette and used in the reference cell of the aggregometer. Test substances (dissolved in saline or 0.2% aqueous DMSO) were added to PRP to a final volume of 300 μl and allowed to incubate for 1 min followed by the addition of an inducer (10 μM ADP or 0.33 U/ml collagen) to initiate aggregation. The maximal gradient of platelet aggregation was defined as the maximal percent increase within the first 5 min. The data were obtained in triplicates. The IC_{50} value was calculated as the concentration of inhibitor causing a 50% inhibition of the aggregation using SPSS software with at least four concentrations.
